# The health care utilisation and out-of-pocket expenditure associated with Australian stroke survivors aged 55 and over

**DOI:** 10.1371/journal.pone.0265907

**Published:** 2022-03-24

**Authors:** David Sibbritt, Mahdie Hosseini, Wenbo Peng, Jessica Bayes, Jon Adams

**Affiliations:** School of Public Health, Faculty of Health, University of Technology Sydney, Sydney, Australia; Monash University Malaysia, MALAYSIA

## Abstract

**Objective:**

Stroke is a major cause of mortality and disability worldwide. People with stroke have a number of options available to treat post-stroke related symptoms and challenges. The aim of this study was to assess the use of healthcare services, self-care practices and out-of-pocket expenses associated with post-stroke healthcare.

**Methods:**

We retrospectively analysed data collected between April and October 2017 from a survey of 576 participants aged 55 to 96 from the 45 and Up Study, NSW (Australia), who had earlier reported a clinical diagnosis of stroke. Participants were asked about their use of health care services, including conventional medicine practitioners and medications, complementary medicine practitioners, practices and products and the respective associated out-of-pocket expenditure for each.

**Results:**

Amongst the total of 576 individuals who participated in the study, 39% consulted a doctor, 18% consulted an allied health practitioner, and 8% consulted a complementary medicine practitioner in the previous year for their stroke. Participants’ average combined out-of-pocket expenditure for post-stroke related healthcare was AU$386.4 per annum. Extrapolated to all Australians with stroke, aged 55 years and over, the total out-of-pocket expenditure for post-stroke related healthcare is estimated to be AU$42 million per annum.

**Conclusions:**

Post-stroke individuals used a wide range of health services and various self-care practices for stroke rehabilitation. Such healthcare utilisation is associated with significant annual out-of-pocket expenditure. Given the socioeconomic burden of stroke, further research is required to identify the barriers and facilitators of self-care among patients with stroke and explore the cost-effectiveness of the wide range of treatments(s) utilised for post-stroke care.

## Introduction

Stroke is one of the major leading causes of disability and death in Australia [1], with more than 445,000 Australians living with the effects of stroke [2]. It has been predicted that by 2050, the number of initial strokes experienced by Australians will increase to 50,600 annually, with 819,900 stroke survivors living in the community [3]. Although the majority of people who experience a stroke are aged 65 years and over, there is an increasing trend in the incidence of stroke in adults younger than 65 years [4]. Research demonstrates that the number of deaths from stroke is declining, resulting in increasing numbers of people who survive and remain significantly disabled from stroke [1].

Despite such improvements in outcomes after acute stroke treatment, most patients experience a wide range of (often chronic) post-stroke challenges such as physical and mental function impairment, limited ability to undertake everyday tasks, restricted social participation as well as decreased quality of life [5, 6] Stroke survivors are typically treated with both medications and rehabilitation services after discharge from hospital [7] and functional recovery from stroke-related health challenges can be a long and costly process [8]. The annual cost of stroke was estimated at $5 billion in Australia in 2013, which includes healthcare costs, informal care and costs associated with loss of productivity [4].

In Australia, outpatient services can either be accessed privately and subsidised through private health insurance or paid directly out of pocket. Publicly funded options also exists where rehabilitation is accessed and co-ordinated through general medical practitioner referrals and subsidised through Medicare [9]. Although most neurologic and function recovery often occurs within a few months following a stroke, the process of recovery may stop permanently, and complete recovery is not experienced by all patients [10].

In addition to practitioner-prescribed treatments, post-stroke individuals are likely to adopt several ‘self-care’ behaviours with a view to helping maintain and comply with prescribed treatments and improve quality of life [11]. Self-care is defined as a set of health behaviours and activities performed by individuals to support health and wellbeing [12]. Established self-care activities important for post-stroke rehabilitation include lifestyle behaviours modification such as smoking cessation, increased physical activity, adopting a healthy diet, and stress reduction [11, 13]. Health professionals have an important role in facilitating and encouraging self-care approaches through information-sharing, promoting patients’ self-efficacy, and providing relevant feedback during the rehabilitative process [11, 14]. Studies suggest stroke patients receiving care from multidisciplinary rehabilitation teams (including nurses, physiotherapists, speech therapists, social workers, and others) have lower mortality, less disability, and improved outcomes compared to those not receiving such care [15, 16].

There also appears to be a growing interest amongst stroke survivors in post-stroke care as to how complementary medicine (CM) may help improve functions during neural recovery [16, 17]. The World Health Organisation (WHO) defines CM as a broad set of healthcare practices that are not fully integrated with the dominant health care system [18]. Some types of CM use in adjunctive care for patients with stroke can be categorized as modalities (e.g. acupuncture and massages) and products (e.g. nutritional supplements and herbal products) [17, 19, 20]. Although research evidence of the efficacy and/or effectiveness of CM therapies/products is limited in post-stroke care, systematic reviews have highlighted the potential effectiveness of traditional Chinese medicines and Tai Chi/Qi Gong use in modifying risk factors of stroke for prevention [20–22].

A wide range of health care services currently exist to help stroke survivors address ongoing post-stroke symptoms and challenges, yet no research to date has analysed the broad use of these healthcare and self-care approaches undertaken by stroke survivors aged 55 years and over living in the community. Therefore, this study aims to identify and assess the utilisation of different health care services, both practitioner led and self-care practices, amongst Australian post-stroke men and women with a particular focus on the out-of-pocket expenditure associated with such use. The healthcare services analysed within this study have been split into the following categories: conventional medicine, which includes medical doctors, allied health practitioners and pharmaceutical medications; CM practitioners; and CM practices and products.

## Materials and methods

### Sample

Data were obtained from a sub-study of the Sax Institute’s 45 and Up Study. The 45 and Up Study baseline questionnaire collected information from 267,153 men and women aged 45 and above who resided in the State of New South Wales, Australia. The 45 and Up Study is described in detail elsewhere [23]. In short, participants were randomly sampled from Services Australia (formerly the Australian Government Department of Human Services) Medicare enrolment database, which offers good representation of the Australian population. There was oversampling of residents from rural and residential areas as well as people aged 80+ years Participants enrolled into the study by completing a baseline questionnaire (January 2006 to December 2009) and providing signed consent for follow-up. The sample represents about 11% of the New South Wales population aged 45 years and over with a response rate of about 18%. The 45 and Up Study was approved by the University of New South Wales Human Research Ethics Committee.

This paper reports data on from a sub-study survey of participants from this cohort, which was administered between April and October 2017. Only participants who had previously indicated on the baseline 45 and Up Study questionnaire that a doctor had diagnosed them as having a stroke were eligible for this sub-study. Invitation to participate in the sub-study was conducted by the Coordinating Centre of the 45 and Up Study in order to provide ethical communications with participants, and the identifiable information (e.g. name and contact details) of the sub-study participants were not released to the sub-study research team. A total of 1,300 45 and Up Study participants who met our inclusion criteria were contacted and mailed a sub-study questionnaire to collect data including demographics information, healthcare utilisation, and stroke status, with 576 (44.3%) returning a completed questionnaire. That is, all these 576 participants indicated that they had a stroke. Ethical approval for this sub-study of 45 and Up Study was gained from the Human Research Ethics Committees at the University of Technology Sydney (approval number: ETH171180). All participants have given their written informed consent prior to their inclusion in this sub-study.

### Demographic characteristics

Participants were asked a number of demographic questions in the sub-study survey. This included information about their gender, current marital status, date of birth, how well they are able to manage on their available income (i.e. no or little difficulty, some difficulties, struggled), their educational qualifications, and if they have health insurance. Based on each participant’s postcode, area of residence was assigned according to the Accessibility Remoteness Index of Australia Plus score (i.e. major city, inner regional area, outer regional or remote area) [24].

### Healthcare utilisation

Participants were asked to report if they had consulted with any of four types of conventional medical practitioners in the previous 12 months and report the frequency of each consultation. These included general practitioners, cardiologists, neurologist, and hospital doctors. They were also asked to specify if they had consulted with any of eight types of allied health practitioners in the previous 12 months and report the frequency of each consultation. These included occupational therapists, physiotherapists, nurses, pharmacist/chemists, psychologists, counsellors, dietitians and speech pathologist. Participants were also provided with a list of 12 groups of CM practitioners and asked to indicate if they consulted with any of these practitioners for their stroke in the previous 12 months. These included traditional Chinese medicine practitioners, acupuncturist, chiropractor, osteopath, massage therapist, naturopath/herbalist, nutritionist, homoeopath, meditation instructor, tai chi instructor, yoga instructor, and an ‘other’ practitioner option).

Participants were also asked to list any prescription medications they had used for their stroke during the previous 12 months. In addition, participants were provided with a list of 22 types of CM products/practices (ginkgo, St Johns wort, herbal medicines, garlic, caffeine-based products or drinks, Coenzyme Q10, multi B vitamin, multivitamins/minerals, folic acid, vitamin B12, vitamin C, vitamin D, vitamin E, omega 3/fish oil, homeopathic remedies, meditation by yourself [i.e. without instructor], mindfulness, physical activities/exercises, [hypericum], Tai Chi by yourself [i.e. without instructor], yoga by yourself [i.e. without instructor], and two ‘other’ CM products/practices options) and asked if they have used any of these products/practices for their stroke during the previous 12 months.

### Out of pocket costs

Regarding the above healthcare services and products for stroke, participants were asked to report the expenses they incurred (out-of-pocket expense) to consult with each service, undertake each of the CM practices; and the purchasing of CM products or prescription medications for their stroke during the previous 12 months. For each group of healthcare providers (i.e. medical/allied health professional, complementary health practitioners) participants were asked ‘How much did it cost you in total for these consultations during the past 12 months?’ For the prescription medications, participants were asked ‘How much did it cost you in total for these medications listed in Question 25 during the past 12 months?’ For the CM products and practices, participants were asked ‘How much did it cost you in total for these products and practices during the past 12 months? The index year for the cost estimates is 2016.

### Stroke status

Information on the time (years/months) since the participants were first diagnosed with stroke was collected. Participants were also asked to rate their degree of disability or dependence that occurred during their daily activities using the modified Rankin Scale (mRS) [25]. The Modified Fatigue Impact Scale—5-item version (MFIS-5) was used to measure their levels of fatigue [26].

### Statistical analyses

A chi-square test was used to examine the association between two categorical variables. Student’s t-test was used to make comparisons between a continuous variable and a categorical variable. Spearman’s correlation coefficient was used to examine the association between two continuous variables. Out-of-pocket expenditure represents self-reported cash payments for health services and related items not covered by Medicare or private health insurance [27]. These costs were categorized as ‘up to $100’, ‘$100-$499’, ‘$500-$999’, ‘$1000-$1499’, and ‘$1500 or above’. All analyses were conducted using the statistical software STATA, version 14. The statistical level of significance was set at 0.05.

## Results

### Demographic characteristics

The average age of respondents was 75.8 (SD = 9.1) years. There were more males (54.9%) than females (45.1%). Almost half of the participants (52.2%) resided in a major city, and the majority of the participants (63.3%) were married or in a de facto relationship. A total of 18.9% of the participants held a university degree, while 32.8% held a certificate or diploma, 35.9% achieved a high school education, and 12.4% had no formal education. When asked how well they can manage on their available income, 66.5% had no or little difficulty, 21.5% had some difficulties, and 12.0% found it difficult to manage. Most of the participants (60.7%) had private health insurance. The average time since the most recent stroke was 10.4 (SD = 8.9) years and all were more than 12 months post-stroke. The majority of participants (73.2%) have had only 1 stroke in total, with 16.1% having had 2 strokes and 10.7% having had 3 or more strokes. In terms of the degree of disability, 85.6% of participants with stroke achieved a good outcome (mRS score 0–2). In terms of fatigue, the average MFIS-5 score was 4.2 (SD = 4.2), with 91.2% having low levels of fatigue (MFIS-5 score ≤10).

### Consultations with healthcare practitioners

A total of 43.9% (n = 253) consulted at least one healthcare practitioner in the previous 12 months for their stroke. A total of 38.5% (n = 222) consulted with a medical doctor, 18.2% (n = 105) consulted with one of the listed allied health practitioners, and 8.0% (n = 46) consulted with a CM practitioner in the previous 12 months.

Table 1 outlines the number of consultations with different healthcare practitioners by stroke characteristics over the past 12 months. Participants who had been diagnosed with stroke for less than 10 years had a larger number of consultations with healthcare practitioners, specifically doctors (p<0.05), compared to participants who had been diagnosed with stroke for 10 years or more. In addition, for the participants who rated their degree of disability as a result of their stroke as being 3–5 points (out of 5) had of more consultations with all types of healthcare practitioners (p<0.05), compared to participants who rated their degree of disability as less than 3 points. In total, our participants had an average of 3.9 consultations with healthcare practitioners in the previous 12 months specifically for their stroke.

**Table 1:** Consultations with health care practitioners over the past 12 months by stroke characteristics.

			Average number of consultations			
Stroke characteristics			Doctor	Allied health practitioner	CM practitioner	Total
			**Mean (SD)**	**Mean (SD)**	**Mean (SD)**	**Mean (SD)**
**Years since**	<10 years	(n = 319)	2.4 (3.5)	1.3 (3.6)	0.7 (2.6)	4.4 (6.8)
**diagnosis**	≥10 years	(n = 257)	1.6 (3.3)	1.2 (4.0)	0.5 (2.0)	3.2 (7.3)
	p-value		0.002	0.569	0.394	0.038
**Degree of**	0–2 points	(n = 374)	1.3 (2.5)	0.6 (2.3)	0.4 (1.9)	2.4 (4.9)
**disability** ^**1**^	3–5 points	(n = 202)	3.4 (4.5)	2.5 (5.3)	0.9 (3.0)	6.7 (9.3)
	p-value		<0.001	<0.001	0.045	<0.001
**Level of**	≤10 points	(n = 516)	2.0 (3.3)	1.2 (3.6)	0.6 (2.3)	3.7 (6.8)
**fatigue** ^**2**^	>10 points	(n = 60)	2.7 (4.4)	1.8 (4.7)	0.8 (3.0)	5.4 (8.8)
	p-value		0.106	0.202	0.513	0.091
**Total**		(n = 576)	2.1 (3.5)	1.3 (3.8)	0.6 (2.3)	

^1^ modified Rankin Scale (mRS) (0 = no disability and 5 = severe disability)

^2^ Modified Fatigue Impact Scale– 5-item version (MFIS-5)

CM: complementary medicine

### Use of prescription medications

Table 2 presents the different prescription medications used by level of fatigue and disability and years since diagnosis. Three categories of prescription medications were determined based on the information provided by participants with stroke: 1) lipid reducing medications such as Lipitor and Atorvastatin. 2) antihypertensives such as Coversyl and Atacand, and 3) blood thinning, anticoagulant or antiplatelet medications such as Warfarin, Plavix and Cartia,. A higher percentage of participants who had been diagnosed with stroke for less than 10 years used a lipid-lowering agent, compared to participants who had been diagnosed with stroke for 10 years or more (p<0.05).

**Table 2:** Use of prescription medications over the past 12 months by stroke characteristics.

			Prescription medication							
Stroke characteristics			Lipid lowering agent ^A^		Antihypertensive ^B^		Anticoagulant or antiplatelet ^C^		Total	
			Yes	No	Yes	No	Yes	No	Yes	No
			(n = 116)	(n = 460)	(n = 208)	(n = 368)	(n = 272)	(n = 304)	(n = 247)	(n = 329)
			**%**	**%**	**%**	**%**	**%**	**%**	**%**	**%**
**Years since**	<10 years	(n = 319)	63.8	53.3	55.7	55.2	59.6	51.6	57.5	52.6
**diagnosis**	≥10 years	(n = 257)	36.2	46.7	44.3	44.8	40.4	48.4	42.5	47.4
	p-value		0.041		0.888		0.056		0.250	
**Degree of**	0–2 points	(n = 374)	67.2	64.4	63.0	66.0	68.0	62.2	66.3	63.2
**disability** ^**1**^	3–5 points	(n = 202)	32.8	35.6	37.0	34.0	32.0	37.8	33.7	36.8
	p-value		0.559		0.461		0.142		0.440	
**Level of**	≤10 points	(n = 516)	93.1	88.7	90.9	88.9	90.1	89.1	90.6	88.3
**fatigue** ^**2**^	>10 points	(n = 60)	6.9	11.3	9.1	11.1	9.9	10.9	9.4	11.7
	p-value		0.165		0.449		0.716		0.367	

^A^ lipid lowering agent such as Atorvastatin, Caduet, Lipitor, and Rosuvastatin.

^B^ antihypertensive such as Atacand, Avapro, Coversyl, and Micardis.

^C^ anticoagulant or antiplatelet such as Cartia, Plavix, Pradaxa, and Warfarin.

^1^ modified Rankin Scale (mRS) (0 = no disability and 5 = severe disability)

^2^ Modified Fatigue Impact Scale– 5-item version (MFIS-5)

### Use of CM products and practices

The use of different CM practices and products used over the previous 12 months is displayed in Table 3. Participants with a higher degree of disability (3–5 points) used CM products or practices less often than those with a lower degree of disability (1–2 points) (p<0.05). No statistically significant associations were found between the year since stroke diagnosis and the number of different CM products and practices used. Additionally, no associations were found between number of products used and level of fatigue.

**Table 3:** Use of complementary medicine products and practices over the past 12 months by stroke characteristics.

			Number of CM products and practices used				
Stroke characteristics			None	1	2	3 or more	p-value
			(n = 343)	(n = 68)	(n = 63)	(n = 102)	
			**%**	**%**	**%**	**%**	
**Years since**	<10 years	(n = 319)	53.1	63.2	55.6	57.8	0.441
**diagnosis**	≥10 years	(n = 257)	46.9	36.8	44.4	42.2	
**Degree of**	0–2 points	(n = 374)	69.1	55.9	66.7	58.9	0.032
**disability** ^**1**^	3–5 points	(n = 202)	30.9	44.1	33.3	44.1	
**Level of**	≤10 points	(n = 516)	90.9	88.2	92.1	84.3	0.233
**fatigue** ^**2**^	>10 points	(n = 60)	9.1	11.8	7.9	15.7	

^1^ modified Rankin Scale (mRS) (0 = no disability and 5 = severe disability)

^2^ Modified Fatigue Impact Scale– 5-item version (MFIS-5)

CM: complementary medicine

### Out-of-pocket expenses

Table 4 presents the out-of-pocket expenses over the past 12 months by stroke characteristics. Participants who had been diagnosed with stroke for less than 10 years had more out-of-pocket costs (p<0.05), particularly for doctors and allied health practitioners, when compared to participants who had been diagnosed with stroke for 10 years or more. Similarly, participants with a higher stroke disability rating (3–5 points) had greater out-of-pocket costs (p<0.05), particularly for doctors and allied health practitioners, compared to participants who rated their degree of stroke disability as being low (1–2 points).

**Table 4:** Out-of-pocket expenses over the past 12 months by stroke characteristics.

			Average cost				
Stroke characteristics			Doctor / allied health practitioner	CM practitioner	Prescription medications	CM products and practices	Total
			**Mean (SD)**	**Mean (SD)**	**Mean (SD)**	**Mean (SD)**	**Mean (SD)**
**Years since**	<10 years	(n = 319)	$174.3 (313.9)	$26.9 (121.5)	$169.7 (273.7)	$79.0 (184.4)	$450.0 (570.1)
**diagnosis**	≥10 years	(n = 257)	$74.7 (224.9)	$32.1 (142.1)	$131.1 (233.2)	$69.4 (189.3)	$307.9 (492.1)
	p-value		<0.001	0.640	0.073	0.542	0.002
**Degree of**	0–2 points	(n = 374)	$85.3 (202.0)	$23.9 (123.7)	$144.6 (234.2)	$66.4 (167.5)	$320.3 (444.5)
**disability** ^**1**^	3–5 points	(n = 202)	$212.4 (375.6)	$39.1 (143.4)	$167.1 (294.4)	$90.1 (217.1)	$508.7 (668.7)
	p-value		<0.001	0.185	0.318	0.147	<0.001
**Level of**	≤10 points	(n = 516)	$125.4 (273.9)	$28.8 (132.3)	$146.6 (246.3)	$72.5 (183.7)	$373.3 (526.9)
**fatigue**^**2**^	>10 points	(n = 60)	$168.3 (343.9)	$33.3 (120.6)	$203.3 (332.7)	$94.2 (209.9)	$499.2 (643.1)
	p-value		0.264	0.799	0.105	0.395	0.088
**Total**		(n = 576)	$129.9 (281.9)	$29.3 (131.0)	$152.5 (256.9)	$74.7 (186.5)	$386.4(540.9)

^1^ modified Rankin Scale (mRS) (0 = no disability and 5 = severe disability)

^2^ Modified Fatigue Impact Scale– 5-item version (MFIS-5)

CM: complementary medicine

Over the past 12 months, the average total healthcare out-of-pocket expenditure by our study participants with stroke was AU$386.4 per annum. In Australia, in 2017, there were 6,634,785 persons aged 55 years and over with an estimated 108,000 (2%) having experienced a stroke (excluding Transient Ischemic Attack) [28]. Extrapolating from these numbers, we estimate the total out-of-pocket expenditure to be approximately AU$42 million per annum for post-stroke related healthcare.

## Discussion

This paper reports the first study to provide a comprehensive analysis of different types of healthcare utilisation and self-care practices for the treatment of post-stroke related symptoms and challenges, and to estimate the associated out-of-pocket expenses of such utilisation. Participants in our study had, on average, 4 consultations with healthcare practitioners over the previous 12 months, with an average out-of-pocket expenditure of AU$386.4 per annum. It is worth noting that our results show more than half of participating stroke survivors did not consult any healthcare practitioner over the past 12 months. In fact, following a stroke, individuals may develop different degrees of disability that can be addressed by consulting healthcare professionals and hence achieve an improved quality of life, independence, and a decrease in the long-term economic cost of stroke [5, 28]. As such, identifying barriers to accessing healthcare services amongst stroke survivors is important for efficient care and secondary stroke prevention.

Our results demonstrate that post-stroke individuals seek more healthcare via doctors, allied health practitioners and/or CM practitioners when they are more disabled. Previous research supports this finding that the level of post-stroke disability experienced is highly associated with health services used by stroke survivors [29, 30]. Such higher usage of healthcare resources may suggest that disability through stroke has unique challenges. Hence, it is important for those managing and organising healthcare provision and services to plan for facilitating rehabilitation in patients with stroke-related disabilities through education or promoting self-care activities, which may result in a reduction in healthcare costs.

In this present study, most participants employed a variety of prescription medications over the past 12 months regardless of their degree of disability experienced, number of years since diagnosis or their level of fatigue. In fact, previous studies have suggested the important role of pharmacotherapies in secondary stroke prevention. For example, most guidelines recommend the use of antiplatelets in all patients with a history of ischaemic stroke; lipid-lowering agents like statins for reducing the risk of recurrent ischaemic stroke irrespective of the cholesterol level; and blood pressure control with antihypertensive drugs for secondary prevention in patients with haemorrhagic stroke [31–34]. However, polypharmacy has the potential for adverse drug effects, drug interactions, greater costs, non-adherence to therapeutic regimens, and to negatively affect the rehabilitation of stroke patients [35, 36]. As such, future investigations should focus on the appropriate polypharmacy use in post-stroke individuals with different types of stroke.

Our study is the first providing an estimate for out-of-pocket expenditure for post-stroke care in Australian people aged 55 years in regard to distinct sectors and types of healthcare. Our results also revealed that participants with less years since stroke diagnosis and major stroke-associated disabilities had greater out-of-pocket expenditure; in particular for the services provided by doctors and allied health practitioners. This is similar to findings from the US which found that patients who were discharged from hospital with major stroke disabilities incurred much higher healthcare costs than those without [37]. Thus, future research should be undertaken to identify the cost-effectiveness of various medical/allied health treatments for post-stroke symptoms and explore the challenges that affect the out-of-pocket costs attributed to post-stroke care.

This study utilised widely used, validated instruments to measure key variables in our analyses and is nested within the largest ongoing cohort study of healthy ageing in the Southern Hemisphere. However, there are some limitations to our study that need to be taken into consideration when interpreting these findings. The results from our study are from a high socio-economic country, where there is an assumption about an initial level of care in the acute phase of stroke. Therefore, these results may differ significantly to other nations and caution should be taken when generating these findings to wider populations. The cohort data is based on respondents’ self-report and may have the potential for recall bias, particularly in health resource utilisation, the scale of stroke-related disability and level of fatigue, and the estimated healthcare expenses. Another limitation concerns the extrapolation to estimate the out-of-pocket expenses for the larger population of Australian with a stroke aged 55 years old and over, which may vary to some extent from the current sample. Similarly, the health-care seeking behaviour of other individuals with stroke might vary from that of the respondents providing the study data. Despite these limitations, this study is the first to comprehensively explore the health service utilisation and self-care practices of people after having a stroke and may direct future health economics research and policy discussions on this topic.

## Conclusion

Our study provides the first data to report a comprehensive analysis of all sectors of healthcare utilisation and the estimated associated out-of-pocket expenses among a sample of individuals with stroke. Stroke survivors are found to have used a wide range of practitioner-led health services and self-care recover from stroke-related symptoms and challenges. Such post-stroke health-seeking behaviour is associated with significant annual out-of-pocket expenditure. Given the high prevalence and socioeconomic burden of post-stroke care, there is an urgent need for further intervention programs addressing specific barriers and facilitators of healthcare among post-stroke individuals, as well as the potential cost-effective treatment(s).
